# Patients Aged ≥55 Years With Stage T1-2N1M1 Differentiated Thyroid Cancer Should Be Downstaged in the Eighth Edition AJCC/TNM Cancer Staging System

**DOI:** 10.3389/fonc.2019.01093

**Published:** 2019-10-18

**Authors:** Zeming Liu, Sichao Chen, Yihui Huang, Di Hu, Min Wang, Wei Wei, Chao Zhang, Wen Zeng, Liang Guo

**Affiliations:** ^1^Department of Plastic Surgery, Zhongnan Hospital of Wuhan University, Wuhan, China; ^2^Department of Pediatrics, St. John Hospital and Medical Center, Detroit, MI, United States; ^3^Department of Cardiovascular Surgery, Tongji Medical College, Union Hospital, Huazhong University of Science and Technology, Wuhan, China; ^4^Department of Ophthalmology, Zhongnan Hospital of Wuhan University, Wuhan, China

**Keywords:** differentiated thyroid cancer, prognosis, SEER, cancer stage, AJCC/TNM

## Abstract

**Objectives:** Since the eighth edition of the American Joint Committee on Cancer tumor-node-metastasis (AJCC/TNM) cancer staging system introduced some significant changes, we investigated whether patients with stage T1-2N1M1 differentiated thyroid cancer (DTC) should be placed in stage IVB, with the goal of providing suggestions for improved survival prediction.

**Materials and Methods:** We divided 30,234 DTC patients aged ≥55 years enrolled from the Surveillance, Epidemiology, and End Results (SEER) database into different stage groups based on the new stage system but in a more thorough manner. Univariate and multivariate Cox regression analyses were conducted to explore the clinicopathological factors associated with cancer-specific survival. Survival of different stage groups was assessed by mortality rates per 1,000 person-years, Cox proportional hazards regression analyses, and Kaplan-Meier analyses with log-rank tests and the propensity score matching method.

**Results:** Univariate and multivariate analyses demonstrated that age at diagnosis, T stage, lymph node metastasis, distant metastasis, histological types, extrathyroidal extension, and radiation therapy were associated with cancer-specific survival. Patients with stage T1-2N1M1 had a lower cancer-specific mortality rate per 1,000 person-years (28.081, 95% confidence interval [CI]: 12.616–62.505) and all-cause mortality rate per 1,000 person-years (70.203, 95% CI: 42.323–116.448) than those with low-level stages such as stage T4aN1M0, stage IVA, and stage T1-2N0M1. Cox proportional hazards regression analyses suggested that patients with stage T4bN1M0 belonging to stage IVA (hazard ratio: 2.529, 95% CI: 1.018–6.278, *p* = 0.046) had a significantly higher risk of cancer-specific mortality than those with stage T1-2N1M1. Kaplan-Meier analyses with log-rank tests suggested that the cancer-specific survival curve of patients with stage T1-2N1M1 had a more modest decline than that of stage T4bN1M0 (*p* = 0.0125), and the cancer-specific survival curve and all-cause survival curve of patients with stage T1-2N1M1 were not different from those of stage T3N1M0, stage T4aN0M0, stage T4aN1M0, stage T4bN0M0, and stage T1-2N0M1 (all, *p* > 0.05). The analysis yielded similar results after propensity score matching for other clinicopathological characteristics.

**Conclusion:** Patients aged ≥55 years with stage T1-2N1M1 DTC according to the eighth edition AJCC/TNM cancer staging system should be downstaged and those with stage T4bN1M0 upstaged accordingly.

## Introduction

Thyroid cancer is the most common malignant endocrine cancer, and its incidence has rapidly increased in the world in recent years (1, 2). Differentiated thyroid cancer (DTC), composed of papillary thyroid cancer (PTC) and follicular thyroid cancer, constitutes almost 86% of all thyroid cancer cases (3, 4).

There are a variety of cancer staging systems for DTC, all of which are aimed at discriminating among different prognostic groups. For example, the 10-year cancer-specific survival (CSS) rate for stage I based on the eighth edition of the American Joint Committee on Cancer tumor-node-metastasis (AJCC/TNM) cancer staging system is almost 99%. In contrast, the 10-year CSS rates for stages IVB and IVC range from 64% to 73% (5, 6). The eighth edition of AJCC/TNM cancer staging system (AJCC/TNM-8) was introduced in clinical practice on January 1, 2017. In this revision, the age cutoff value was revised from 45 to 55 years, and the descriptors of the T and N stages were changed from those contained in the seventh edition (7, 8). As a result, nearly 30% of DTC patients were downstaged following the introduction of the new edition (8).

Several surveys about the new TNM classification system have indicated that its prognostic value for survival is better than that of the seventh edition (5, 9). However, it is unclear whether the prognosis of DTC patients worsens with increasing stage; thus, it is important to thoroughly evaluate how well the new cancer staging system edition correlates with the survival of DTC patients. As is well known, age at diagnosis is considered an independent predictor of DTC prognosis, and it does not significantly influence CSS until patients are aged 50–60 years (10–12). Taking this into consideration, older DTC patients with poorer prognoses require more accurate and detailed cancer staging. The study aimed to refine the new TNM cancer staging system for older DTC patients.

## Materials and Methods

### Data Collection

The protocol of this retrospective study was approved by the Ethics Review Board of Zhongnan Hospital of Wuhan University. The need for informed consent was waived because of the retrospective nature of the study. The Surveillance, Epidemiology and End Results (SEER) database of the National Cancer Institute (https://seer.cancer.gov/), which contains demographic, pathological and treatment characteristics on cancer patients, was used as the source of data. We recruited patients aged ≥55 years with DTC from the Surveillance, Epidemiology, and End Results (SEER) database of the National Cancer Institute (https://seer.cancer.gov/) by using its official software “SEERStat” version 8.3.4. and code C73.9 from the International Classification of Diseases for Oncology (i.e., thyroid, papillary). The eligible diagnoses were as follows: “papillary carcinoma,” “papillary adenocarcinoma,” “follicular carcinoma,” “follicular adenocarcinoma,” “papillary carcinoma, follicular variant,” and “papillary & follicular adenocarcinoma.” Patients with missing data on survival or AJCC staging information (version 8) were excluded. In total, this study included 30,234 patients with DTC from 2004 to 2013. All included patients were followed up until December 2013, and the median follow-up time was 42 months. The median follow-up time of each staging group is listed in the Supplementary Materials.

### More Detailed Cancer Staging Groups

Based on the AJCC/TNM-8 categories, patients were divided into stage I, stage II, stage III, stage IVA, and stage IVB. For a more detailed evaluation of the ability to predict survival, stage II patients were divided into stage T1N1M0, stage T2N1M0, stage T3N0M0, and stage T3N1M0; stage III patients were divided into stage T4aN0M0 and stage T4aN1M0; stage IVA patients were divided into stage T4bN0M0 and stage T4bN1M0; and stage IVB patients were divided into stage T1-2N0M1, stage T1-2N1M1, stage T3-4N0M1, and stage T3-4N1M1.

### Statistical Analysis

Quantitative variables are expressed as median (interquartile range), while categorical variables are presented as percentages. We explored the clinicopathological factors associated with cancer-specific mortality (CSM) using univariate and multivariate Cox regression analyses and compared the CSM and all-cause mortality (ACM) rates per 1,000 person-years of the same or adjacent AJCC/TNM-8 stage groups. Cox proportional hazards regression analyses were then used to assess the effect of the different stage groups on CSM and ACM, with adjustment for demographic, pathological, and treatment characteristics. Finally, Kaplan-Meier analyses with log-rank tests were performed with propensity score matching applied to minimize selection bias. Moreover, the relative excess risk (RERI), attributable proportion (AP), and synergy index (SI) were used to evaluate the synergic effect.

All *p*-values were two-sided, with *p* < 0.05 considered statistically significant. Statistical analyses were performed using SPSS version 24.0 (IBM Corp., Armonk, NY), GraphPad Prism version 6 (GraphPad Software Inc., La Jolla, CA) and Stata/SE version 15 (Stata Corp., College Station, TX).

## Results

### Demographic and Clinical Characteristics

The demographic and clinical characteristics of 30,234 patients with DTC enrolled in this study are summarized in Table 1. According to the AJCC/TNM-8 classification system, 22,925 patients (75.8%) were classified as stage I; 4,977 (16.5%) stage II patients included 2,370 patients with stage T1-2N1M0, 2,109 patients with stage T3N0M0, and 498 patients with stage T3N1M0; 1,084 (3.6%) stage III patients included 517 patients with stage T4aN0M0 and 567 patients with stage T4aN1M0; 597 (2.0%) stage IVA patients included 260 patients with stage T4bN0M0 and 337 patients with stage T4bN1M0; and 651 (2.2%) stage IVB patients included 139 patients with stage T1-2N0M1, 56 patients with stage T1-2N1M1, 175 patients with stage T3-4N0M1, and 281 patients with stage T3-4N1M1. In addition, while 1,999 (6.6%) patients had T4 tumors, 4,109 (13.6%) patients had lymph node metastasis (LNM), and 651 (2.2%) patients had distant metastasis (DM).

**Table 1:** Demographic and clinicopathological characteristics of 30,234 patients with DTC.

**Characteristics**	**Number (%)**
**Age at diagnosis (year)**	
Median (interquartile range)	64 (59–70)
**Year of diagnosis**	
2004–2008	11,952 (39.5)
2009–2013	18,282 (60.5)
**Sex**	
Female	21,393 (70.8)
Male	8,841 (29.2)
**Race**	
White	25,108 (83.7)
Black	2,152 (7.2)
Other	2,750 (9.1)
**AJCC Staging Grouping (8th Edition)**	
Stage at diagnosis I	22,925 (75.8)
Stage at diagnosis II	4,977 (16.5)
T1-2N1M0	2,370 (7.8)
T3N0M0	2,109 (7.0)
T3N1M0	498 (1.7)
Stage at diagnosis III	1,084 (3.6)
T4aN0M0	517 (1.7)
T4aN1M0	567 (1.9)
Stage at diagnosis IVA	597 (2.0)
T4bN0M0	260 (0.9)
T4bN1M0	337 (1.1)
Stage at diagnosis IVB	651 (2.2)
T1-2N0M1	139 (0.5)
T1-2N1M1	56 (0.2)
T3-4N0M1	175 (0.6)
T3-4N1M1	281 (0.9)
**T stage at diagnosis**	
T1	20,749 (68.6)
T2	4,741 (15.7)
T3	2,745 (9.1)
T4a	1,231 (4.1)
T4b	768 (2.5)
Lymph node metastasis	4,109 (13.6)
Distant metastasis	651 (2.2)
Multifocality	11,097 (37.3)
**Histology subtype**	
PTC	28,001 (93.0)
FTC	2,115 (7.0)
Extrathyroidal extension	2,570 (8.5)
**Radiation therapy**	
None or refused	17,353 (58.6)
Radiation beam or Radioactive implants	695 (2.3)
Radioisotopes or Radiation beam plus isotopes or implants	11,559 (39.0)
**Surgery**	
Lobectomy	5,348 (18.3)
Subtotal or near-total thyroidectomy	1,266 (4.3)
Total thyroidectomy	22,679 (77.4)

*DTC, differentiated thyroid cancer; PTC, papillary thyroid cancer; FTC, follicular thyroid cancer*.

### Risk Factors for Cancer-Specific Mortality in DTC

Based on the results of the univariate Cox regression analyses, the CSM of DTC patients was associated with age at diagnosis, year at diagnosis, sex, race, T stage, LNM, DM, histological types, extrathyroidal extension (ETE), radiation therapy, and surgery (all, *p* < 0.001). The multivariate Cox regression analyses demonstrated that the CSM of DTC patients was associated with age at diagnosis, T stage, LNM, DM, histological types, ETE, and radiation therapy (all, *p* < 0.05). Consequently, T stage, LNM, and DM were associated with CSM of DTC patients according to univariate and multivariate Cox regression analyses. The multivariate Cox regression model showed that the CSM rate of DTC patients with T4a or T4b stage tumors was significantly higher than that of patients with T1 stage after adjusting for other clinicopathological characteristics (hazard ratio [HR]: 7.714, 95% confidence interval [CI]: 4.640–12.824; HR: 13.479, 95% CI: 8.094–22.447, respectively). The CSM rate of DTC patients with LNM or DM was also notably higher than that of patients without them (HR: 1.988, 95% CI: 1.608–2.459; HR: 4.670, 95% CI: 3.768–5.789, respectively) (Table 2).

**Table 2:** Clinicopathological parameters associated with the cancer-specific deaths.

**Parameters**		**HR**	**Univariate**			**HR**	**Multivariate**		
			**95% CI**		***p*-value**		**95% CI**		***p*-value**
Age at diagnosis		1.092	1.083	1.101	<0.001^*^	1.055	1.045	1.065	<0.001^*^
Year at diagnosis	2004–2008	Ref				Ref			
	2009–2013	0.716	0.606	0.845	<0.001^*^	0.878	0.719	1.073	0.203
Sex	Female	Ref				Ref			
	Male	1.852	1.595	2.151	<0.001^*^	1.155	0.963	1.385	0.120
Race	White	Ref				Ref			
	Black	0.721	0.510	1.019	0.064	0.651	0.404	1.051	0.079
	Other	1.540	1.238	1.915	<0.001^*^	0.898	0.693	1.164	0.417
T-Stage at diagnosis	T1	Ref				Ref			
	T2	4.039	2.885	5.653	<0.001^*^	2.948	2.002	4.343	<0.001^*^
	T3	13.058	9.724	17.534	<0.001^*^	5.000	3.300	7.576	<0.001^*^
	T4a	49.555	37.726	65.093	<0.001^*^	7.714	4.640	12.824	<0.001^*^
	T4b	111.668	85.632	145.620	<0.001^*^	13.479	8.094	22.447	<0.001^*^
Lymph node metastasis	No	Ref				Ref			
	Yes	8.627	7.432	10.014	<0.001^*^	1.988	1.608	2.459	<0.001^*^
Distant metastasis	No	Ref				Ref			
	Yes	28.798	24.546	33.785	<0.001^*^	4.670	3.768	5.789	<0.001^*^
Multifocality	No	Ref				Ref			
	Yes	0.955	0.813	1.122	0.576	0.845	0.704	1.014	0.070
Histological Types	Papillary	Ref				Ref			
	Follicular	2.191	1.772	2.709	<0.001^*^	1.475	1.117	1.946	0.006^*^
Extrathyroidal extension	No	Ref				Ref			
	Yes	28.771	24.385	33.947	<0.001^*^	2.692	1.780	4.070	<0.001^*^
Radiation	None or refused	Ref				Ref			
	Radiation beam or radioactive implants	17.615	14.482	21.425	<0.001^*^	2.444	1.884	3.171	<0.001^*^
	Radioisotopes or radiation beam plus isotopes or implants	1.415	1.192	1.680	<0.001^*^	0.684	0.552	0.848	0.001^*^
Surgery	Lobectomy	Ref				Ref			
	Subtotal or near total thyroidectomy	2.221	1.505	3.279	<0.001^*^	1.129	0.722	1.765	0.596
	Total thyroidectomy	1.668	1.295	2.147	<0.001^*^	0.995	0.744	1.331	0.974

* *Represent the p-value < 0.05*.

### CSM and ACM Rates per 1,000 Person-Years

During the follow-up until December 2013, the CSM rates per 1,000 person-years for DTC patients of the detailed stage groups are shown in Table 3. The CSM for stages T3N0M0, T3N1M0, T4aN0M0, T4aN1M0, T4bN0M0, and T4bN1M0 gradually increased. However, the CSM for stage T1-2N0M1 (61.051, 95% CI: 41.868–89.024) was even lower than that for stage T4bN1M0 (76.981, 95% CI: 62.238–95.216), and the CSM for stage T1-2N1M1 (28.081, 95% CI: 12.616–62.505) was even lower than that for stage T4aN1M0 (41.667, 95% CI: 33.645–51.602) and stage IVA. Notably, the CSM for stage T4bN1M0 (76.981, 95% CI: 62.238–95.216) was more than twice that for stage T1-2N1M1. Furthermore, during the follow-up period, the ACM rates per 1,000 person-years for patients of these stage groups yielded similar results (Table 3).

**Table 3:** Measures of cancer-specific death and all-cause death in differentiated thyroid cancer.

		**Total number**	**Cancer-specific mortality**	**%**	**Cancer-specific mortality**	**95%CI**	**All-cause mortality**	**%**	**All-cause mortality**	**95% CI**
			**No**.		**1,000 person-years**		**No**.		**1,000 person-years**	
Stage II	T3N0M0	2,109	36	1.7	4.264	3.076–5.911	252	11.9	29.847	26.380–33.769
	T3N1M0	498	45	9.0	26.045	19.447–34.884	93	18.7	53.827	43.928–65.958
Stage III	T4aN0M0	517	57	11.0	26.540	20.472–34.407	112	21.7	52.150	43.333–62.760
	T4aN1M0	567	84	14.8	41.667	33.645–51.602	168	29.6	83.333	71.639–96.937
Stage IVA	T4bN0M0	260	51	19.6	49.671	37.750–65.358	88	33.8	85.707	69.547–105.622
	T4bN1M0	337	85	25.2	76.981	62.238–95.216	129	38.3	116.830	98.313–138.835
Stage IVB	T1-2N0M1	139	27	19.4	61.051	41.868–89.024	47	33.8	106.275	79.849–141.446
	T1-2N1M1	56	6	10.7	28.081	12.616–62.505	15	26.8	70.203	42.323–116.448
	T3-4N0M1	175	57	32.6	126.129	97.291–163.516	80	45.7	177.024	142.188–220.394
	T3-4N1M1	281	117	41.6	194.164	161.985–232.736	171	60.9	283.779	244.279–329.665

### HRs of Different Stage Groups for CSM and ACM Rates

Table 4 displays the HRs for cancer-specific death for stage T1-2N1M1 vs. other stages. The HR of stage T4bN1M0 was 2.837 (95% CI: 1.241–6.484, *p* = 0.013). After adjustment for demographic data (age at diagnosis, year at diagnosis, sex, and race), the HR of stage T4bN1M0 was 2.372 (95% CI: 1.037–5.427, *p* = 0.041). After adjustment for demographic data and pathological factors (multifocality and histological subtypes), the HR of stage T4bN1M0 was 2.623 (95% CI: 1.063–6.476, *p* = 0.036). After adjustment for demographic data, pathological factors, and therapeutic factors (surgery and radiation therapy), the HR of stage T4bN1M0 was 2.529 (95% CI: 1.018–6.278, *p* = 0.046) (Table 4). In addition, all 95% CIs of HRs of the other stage groups (stages T3N1M0, T4aN0M0, T4aN1M0, T4bN0M0, and T1-2N0M1) included 1. Therefore, there were no significant differences in the risk of cancer-specific death between stage T1-2N1M1 and the other lower stages. Similar results were noted for all-cause death, and the HRs for all-cause death for T1-2N1M1 vs. other stages are displayed in Table S1.

**Table 4:** Hazard ratios of AJCC cancer staging (8th edition) for cancer-specific mortality.

**Stage at diagnosis**	**Total number**	**Unadjusted cox regression**		**Adjusted 1 cox regression**		**Adjusted 2 cox regression**		**Adjusted 3 cox regression**	
		**Hazard ratio (95% CI)**	***p*-value**	**Hazard ratio (95% CI)**	***p*-value**	**Hazard ratio (95% CI)**	***p*-value**	**Hazard ratio (95% CI)**	***p*-value**
T3N1M0	498	0.911 (0.388–2.134)	0.829	0.869 (0.370–2.036)	0.746	0.941 (0.372–2.383)	0.899	1.039 (0.407–2.648)	0.937
T4aN0M0	517	1.025 (0.443–2.373)	0.954	0.891 (0.385–2.065)	0.788	0.903 (0.360–2.263)	0.828	0.876 (0.348–2.202)	0.778
T4aN1M0	567	1.506 (0.658–3.445)	0.332	1.321 (0.577–3.025)	0.510	1.423 (0.576–3.515)	0.444	1.372 (0.553–3.405)	0.495
T4bN0M0	260	2.026 (0.873–4.699)	0.100	1.725 (0.743–4.005)	0.205	1.870 (0.746–4.689)	0.182	1.798 (0.714–4.528)	0.213
T4bN1M0	337	2.837 (1.241–6.484)	0.013^*^	2.372 (1.037–5.427)	0.041^*^	2.623 (1.063–6.476)	0.036^*^	2.529 (1.018–6.278)	0.046^*^
T1-2N0M1	139	2.159 (0.894–5.214)	0.087	1.656 (0.684–4.009)	0.264	1.908 (0.735–4.953)	0.184	1.671 (0.642–4.354)	0.293
T1-2N1M1	56	Ref		Ref		Ref		Ref	

*Adjusted 1 Cox regression: cox regression for age at diagnosis, year at diagnosis, sex and race matched subtype pairs*.

*Adjusted 2 Cox regression: cox regression for age at diagnosis, year at diagnosis, sex, race, multifocality and histological subtypes matched subtype pairs*.

*Adjusted 3 Cox regression: cox regression for age at diagnosis, year at diagnosis, sex, race, multifocality and histology, surgery and radiation therapy matched subtype pairs*.

* *Represents p-value < 0.05*.

### Kaplan-Meier Analysis and Propensity Score Matching Method

**Figure 1:**
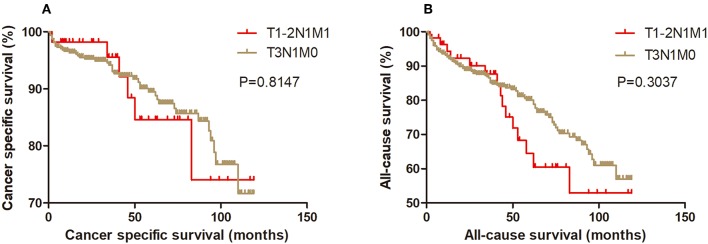
Kaplan Meier curves between older DTC patients with T1-2N1M1 stage and with T3N1M0 stage for cancer-specific mortality **(A)** and all-cause mortality **(B)**.

**Figure 2:**
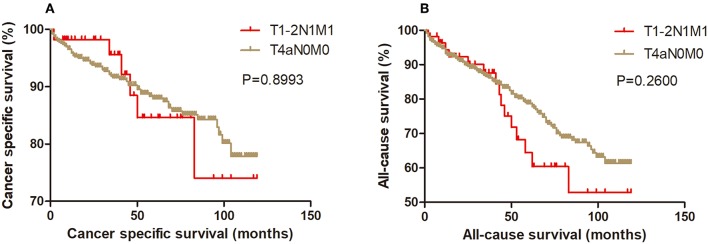
Kaplan Meier curves between older DTC patients with T1-2N1M1 stage and with T4aN0M0 stage for cancer-specific mortality **(A)** and all-cause mortality **(B)**.

**Figure 3:**
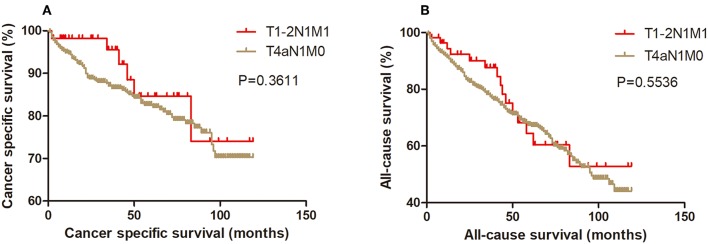
Kaplan Meier curves between older DTC patients with T1-2N1M1 stage and with T4aN1M0 stage for cancer-specific mortality **(A)** and all-cause mortality **(B)**.

**Figure 4:**
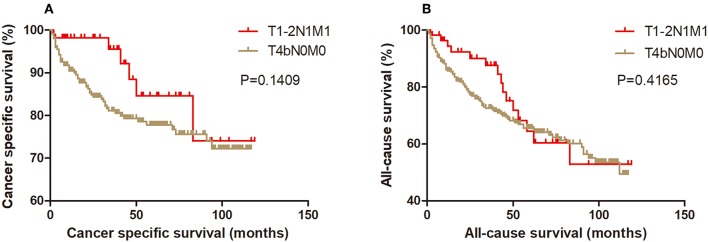
Kaplan Meier curves between older DTC patients with T1-2N1M1 stage and with T4bN0M0 stage for cancer-specific mortality **(A)** and all-cause mortality **(B)**.

**Figure 5:**
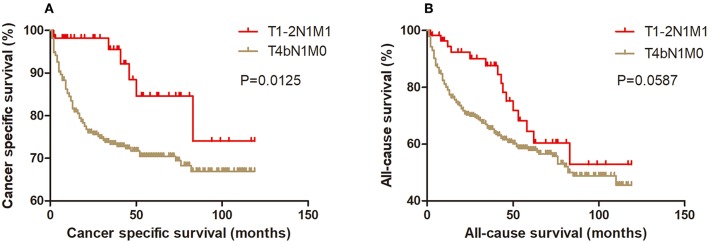
Kaplan Meier curves between older DTC patients with T1-2N1M1 stage and with T4bN1M0 stage for cancer-specific mortality **(A)** and all-cause mortality **(B)**.

**Figure 6:**
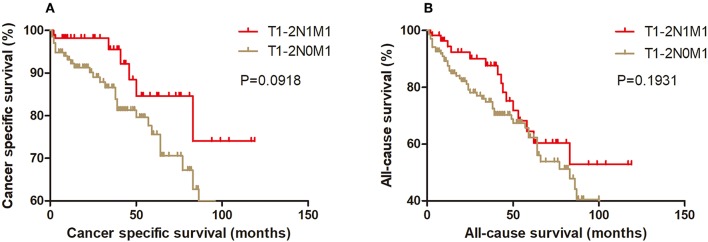
Kaplan Meier curves between older DTC patients with T1-2N1M1 stage and with T1-2N0M1 stage for cancer-specific mortality **(A)** and all-cause mortality **(B)**.

Kaplan-Meier analysis of survival was performed between DTC patients with stage T1-2N1M1 and stages T3N1M0, T4aN0M0, T4aN1M0, T4bN0M0, T4bN1M0, and T1-2N0M1 (Figures 1–6). The CSS curve of patients with T1-2N1M1 stage showed a more modest decline than that of patients with stage T4bN1M0 (Figure 5A). However, analysis of all-cause survival showed a lack of significant difference for the two groups (Figure 5B). In addition, the cancer-specific curve and all-cause curve for stage T1-2N1M1 did not diverge from those of stages T3N1M0, T4aN0M0, T4aN1M0, T4bN0M0, and T1-2N0M1 (Figures 1–4, 6).

Moreover, we used the 1:2 propensity score matching method to match samples of the other six stages that had similar clinicopathological characteristics, including age at diagnosis, year at diagnosis, sex, race, multifocality, histological subtypes, surgery, and radiation, with those of the T1-2N1M1 subgroup. After propensity score matching for age at diagnosis, year at diagnosis, sex and race, the T1-2N1M1 subgroup did not have a significantly different CSM from that of the T3N1M0, T4aN0M0, T4aN1M0, T4bN0M0, T1-2N0M1 subgroups, but had a lower CSM than that of the T4bN1M0 subgroup (*p* = 0.0182) (Figure S1). After propensity score matching for age at diagnosis, year at diagnosis, sex and race, the T1-2N1M1 subgroup did not have a significantly different ACM from that of the T3N1M0, T4aN0M0, T4aN1M0, T4bN0M0, T4bN1M0, and T1-2N0M1 subgroups (Figure S2). After propensity score matching for age at diagnosis, year at diagnosis, sex, race, multifocality and histological subtypes, the T1-2N1M1 subgroup did not have a significantly different CSM or ACM from that of the T3N1M0, T4aN0M0, T4aN1M0, T4bN0M0, T4bN1M0, and T1-2N0M1 subgroups (Figures S3, S4). The analysis yielded similar results when the data were propensity score matched for age at diagnosis, year at diagnosis, sex, race, multifocality, histological subtypes, surgery, and radiation (Figures S5, S6).

### Synergic Effect of T4 Stage and LNM on Survival in DTC

We classified DTC patients into 4 subtypes according to T stage and N stage (T1-3N0, T1-3N1, T4N0, and T4N1). As shown in Table 5, stage T4N1 patients had the largest risk of CSM (HR = 6.076, 95% CI: 4.150–8.895, *p* < 0.001), when compared with CSM of other combinations of T stage and N status and after adjustment for age at diagnosis, year at diagnosis, sex, race, M status, multifocality, histology subtypes, ETE, radiation therapy, and surgery.

**Table 5:** Measures for estimation of synergic effect between T stage and N stage for the cancer-specific mortality of DTC.

**T stage**	**N status**	**Death events (%)**	**Total case**	**HR (95% CI)**	***p*-value**
T1-3	N0	150	25,249	Reference	
T1-3	N1	106	2,986	4.679 (3.476–6.299)	<0.001^*^
T4	N0	163	876	4.658 (3.166–6.854)	<0.001^*^
T4	N1	281	1,123	6.076 (4.150–8.895)	<0.001^*^
RERI	12.430 (2.232–22.630)				
AP	0.223 (0.064–0.381)				
SI	1.293 (1.050–1.592)				

*Adjusted for age at diagnosis, year at diagnosis, sex, race, M status, multifocality, histology subtypes, extrathyroidal extension, radiation, surgery*.

* *Represent the p-value < 0.05; RERI, relative excess risk; AP, attributable proportion; SI, synergy index*.

Based on the abovementioned results, the RERI was 12.430 (95% CI: 2.232–22.630), AP was 0.223 (95% CI: 0.064–0.381), and SI was 1.293 (95% CI: 1.050–1.592) (Table 5).

The synergic effect of T4 stage and LNM on ACM was evaluated, and similar results were found (Table S2).

## Discussion

In this study, we enrolled 30,234 DTC patients aged ≥55 years. Based on the AJCC/TNM-8 system, stage I, stage II, stage III, and stage IV accounted for 75.8, 16.5, 3.6, and 4.2% of patients, respectively. Shaha et al. (8) reported that DTC patients with stage I, stage II, stage III, and stage IV accounted for 84, 13, 2, and 1%, respectively, in their study, while Song et al. (13) reported proportions of 85.9, 12.2, 1.2, and 0.7%, respectively. These discrepancies might be because our age cutoff was older than that used in the other studies; thus, a larger proportion of patients in our study had a higher stage.

More importantly, we observed that DTC patients aged ≥55 years with stage T1-2N1M1 should be downstaged in the AJCC/TNM-8 cancer staging system. In our study, patients with stage T1-2N1M1 had significantly lower DSM and ACM than those with stage T4bN1M0. Moreover, patients with stage T4bN1M0 had a more than 2-fold increased risk of cancer-specific death and all-cause death compared to patients with stage T1-2N1M1. Notably, in the study conducted by Rosario (14), among 19 patients with stage T4M0 who were classified as stage I according to the AJCC/TNM-8, there were 2 deaths among patients with stage T4aN1bM0 and T4bN1bM0 because of PTC. In addition, owing to the uncommon deaths, the author called for more studies that evaluated mortality specifically in T4N1b patients aged 45–55 years.

A good cancer staging system should focus on effective discrimination and prediction of the prognoses of DTC patients. In DTC patients, the prognosis worsens as the stage increases. Minimal ETE is excluded from the definition of stage T3 in AJCC/TNM-8 because it does not lead to higher mortality (15, 16). Since patients with stage T1-2N1M1 had lower mortality than patients with stage T4bN1M0, we suggest that patients with T1-2N1M1 disease be downstaged, and patients with stage T4bN1M0 be upstaged, in terms of better distinguishing the prognoses of different DTC patients.

DM is considered an indicator of compromised survival among DTC patients. Patients with DM are recommended to undergo more aggressive treatments such as total thyroidectomy followed by radioiodine therapy and thyroid-stimulating hormone suppressive therapy (17, 18). However, the aforementioned survival results reveal that patients with stage T4bN1M0 disease should be given more aggressive treatment, even in the absence of DM.

We hypothesized that the worse prognosis of T4bN1M0 stage disease is due to the synergic effect of gross ETE beyond the strap muscles (T4 stage) and LNM in DTC patients aged ≥55 years. The RERI (12.430, 95% CI: 2.232–22.630) indicates that there would be a 12.430 relative excess risk contributed by the additive synergic effect of gross ETE beyond the strap muscles and LNM. The AP (0.223, 95% CI: 0.064–0.381) suggests that 22.3% of the increased risk of CSM was caused by the synergic effect (19). In addition, the SI (1.293, 95% CI: 1.050–1.592) was larger than 1, which also signified the existence of a synergic effect (20). Summarily, the results (RERI > 0, AP > 0, and SI > 1) verified our hypothesis.

Another finding of this study was that the 1,000 person-years CSM of T1-2N1M1 stage patients was notably lower than that of T1-2N0M1 stage patients. However, the results of univariable and multivariable Cox regression analyses suggest that the N stage at diagnosis was associated with the CSS of DTC patients. In other studies, LNM was also associated with decreased survival (21–23).

This contradictory result may be due to the fact that there are other indicators of survival besides N stage in terms of LNM. Pyo et al. conducted a meta-analysis and showed that a high metastatic lymph node ratio (≥0.44) was notably correlated with worse survival of PTC patients (24), while Wei et al. demonstrated that the number of positive lymph nodes could be a better predictor of survival of DTC patients (23). In addition, location played a critical role, and positive lymph nodes in the lateral compartment were correlated with a shorter disease-free survival than those in the central compartment according to de Meer et al. (25). Thus, when evaluating the risk of LNM, clinicians should consider the N stage and the number, ratio, and location of metastasizing lymph nodes.

Our study has some limitations. First, because it was a retrospective study, selection bias could not be avoided. Second, there were only 56 patients with stage T1-2N1M1. Most DTC patients had a good prognosis, and only a small proportion of DTC patients belonged to stage IV. However, we recruited 30,234 DTC patients aged ≥55 years to enroll as many stage IVB patients as possible. Furthermore, the propensity score matching method was utilized to validate our conclusions. However, future clinical trials focusing on this stage group of DTC patients are needed. Third, we did not consider the extent of metastatic disease in the DTC patients in this study due to lack of information.

In conclusion, DTC patients with stage T1-2N1M1 have a better prognosis than those with stage T4bN1M0 and no significantly worse prognosis than those with lower stages. Therefore, they should be downstaged, and patients with stage T4bN1M0 should be upstaged, accordingly.

## Data Availability Statement

Publicly available datasets were analyzed in this study. This data can be found here: https://seer.cancer.gov/.

## Ethics Statement

This study was carried out in accordance with the recommendations of guidelines of the American Thyroid Association. The need for informed consent was waived on account of the retrospective nature of the study. The protocol was approved by the Ethics Review Board of Zhongnan Hospital of Wuhan University.

## Author Contributions

LG, WZ, and ZL contributed the conception and design of the study. YH and DH organized the database. SC performed the statistical analysis and wrote the first draft of the manuscript. MW, WW, and CZ wrote sections of the manuscript. All authors contributed to the manuscript revision, read, and approved the submitted version.

### Conflict of Interest

The authors declare that the research was conducted in the absence of any commercial or financial relationships that could be construed as a potential conflict of interest.

## Abbreviations

DTC: differentiated thyroid cancer
PTC: papillary thyroid cancer
AJCC/TNM: American Joint Committee on Cancer tumor-node-metastasis
AJCC/TNM-8: The eighth edition of AJCC/TNM cancer staging system
SEER: Surveillance, Epidemiology, and End Results
CSS: cancer-specific survival
ACS: all-cause survival
CSM: cancer-specific mortality
ACM: all-cause mortality
RERI: relative excess risk
AP: attributable proportion
SI: synergy index
LNM: lymph node metastasis
DM: metastasis
ETE: extrathyroidal extension.
