# Supplementary material for: Patients Aged ≥55 Years With Stage T1-2N1M1 Differentiated Thyroid Cancer Should Be Downstaged in the Eighth Edition AJCC/TNM Cancer Staging System
Source: Front Oncol. 2019 Oct 18;9:1093. doi: 10.3389/fonc.2019.01093 (PMC6813624; doi:10.3389/fonc.2019.01093)

Supplementary Tables

Table S1: Hazard ratios of AJCC Cancer Staging (8th Edition) for all cause mortality

| Stage at diagnosis | Unadjusted Cox regression | | Adjusted 1 Cox regression | | Adjusted 2 Cox regression | | Adjusted 3 Cox regression | |
| --- | --- | --- | --- | --- | --- | --- | --- | --- |
|  | Hazard Ratio  (95% CI) | p-value | Hazard Ratio  (95% CI) | p-value | Hazard Ratio  (95% CI) | p-value | Hazard Ratio  (95% CI) | p-value |
| T3N1M0 | 0.755(0.438~1.303) | 0.314 | 0.714(0.414~1.232) | 0.226 | 0.738(0.411~1.324) | 0.308 | 0.791(0.439~1.427) | 0.436 |
| T4aN0M0 | 0.793(0.464~1.358) | 0.398 | 0.704(0.410~1.207) | 0.202 | 0.696(0.390~1.241) | 0.220 | 0.675(0.377~1.208) | 0.186 |
| T4aN1M0 | 1.189(0.701~2.016) | 0.520 | 1.020(0.601~1.730) | 0.943 | 1.077(0.611~1.898) | 0.797 | 1.081(0.611~1.912) | 0.788 |
| T4bN0M0 | 1.342(0.779~2.313) | 0.289 | 1.171(0.678~2.021) | 0.572 | 1.156(0.643~2.080) | 0.628 | 1.159(0.642~2.093) | 0.625 |
| T4bN1M0 | 1.718(1.008~2.929) | 0.047* | 1.423(0.834~2.430) | 0.196 | 1.468(0.827~2.607) | 0.190 | 1.436(0.805~2.562) | 0.221 |
| T1-2N0M1 | 1.503(0.842~2.685) | 0.168 | 1.108(0.619~1.983) | 0.730 | 1.223(0.660~2.266) | 0.523 | 1.116(0.600~2.075) | 0.729 |
| T1-2N1M1 | ref |  | ref |  | ref |  | ref |  |

Adjusted 1 Cox regression: cox regression for age at diagnosis, year at diagnosis,sex and race matched subtype pairs.

Adjusted 2 Cox regression: cox regression for age at diagnosis, year at diagnosis, sex, race, , multifocality and histology matched subtype pairs.

Adjusted 3 Cox regression: cox regression for age at diagnosis, year at diagnosis, sex, race, multifocality and histology, surgery and radiation therapy matched subtype pairs.

* represent the p value <0.05.

Table S2 Measures for estimation of synergic effect between T stage and N stage for the all cause mortality of DTC

| T stage | N status | Death events(%) | Total case | HR(95% CI) | p value |
| --- | --- | --- | --- | --- | --- |
| T1-3 | N0 | 1896 | 25249 | reference |  |
| T1-3 | N1 | 345 | 2986 | 1.594(1.401~1.813) | <0.001* |
| T4 | N0 | 273 | 876 | 1.660(1.324~2.080) | <0.001* |
| T4 | N1 | 454 | 1123 | 2.211(1.782~2.743) | <0.001* |
| RERI | 2.173(0.883~3.463) | | | | |
| AP | 0.260(0.128~0.392) | | | | |
| SI | 1.419(1.152~1.747) | | | | |

Note: Adjusted for age at diagnosis, year at diagnosis, sex, race, M status, multifocality, histology subtypes, extrathyroidal extension, radiation, surgery. * represent the p value < 0.05; RERI, relative excess risk; AP, attributable proportion; SI, synergy index.

Table S3: Hazard ratios of AJCC Cancer Staging (8th Edition) for cancer specific mortality

| Stage at diagnosis | Total number | Unadjusted Cox regression | | Adjusted 1 Cox regression | | Adjusted 2 Cox regression | | Adjusted 3 Cox regression | |
| --- | --- | --- | --- | --- | --- | --- | --- | --- | --- |
|  |  | Hazard Ratio  (95% CI) | p-value | Hazard Ratio  (95% CI) | p-value | Hazard Ratio  (95% CI) | p-value | Hazard Ratio  (95% CI) | p-value |
| T3N1M0 | 498 | ref |  | ref |  | ref |  | ref |  |
| T4aN0M0 | 517 | 1.126(0.765~1.657) | 0.548 | 1.048(0.710~1.548) | 0.814 | 0.983(0.650~1.487) | 0.936 | 0.861(0.562~1.318) | 0.490 |
| T4aN1M0 | 567 | 1.654(1.153~2.372) | 0.006 | 1.527(1.064~2.192) | 0.022 | 1.567(1.073~2.287) | 0.020 | 1.343(0.903~1.999) | 0.145 |
| T4bN0M0 | 260 | 2.224(1.504~3.289) | <0.001 | 2.021(1.361~3.000) | <0.001 | 2.011(1.326~3.051) | 0.001 | 1.754(1.138~2.705) | 0.011 |
| T4bN1M0 | 337 | 3.116(2.178~4.456) | <0.001 | 2.763(1.928~3.961) | <0.001 | 2.880(1.976~4.197) | <0.001 | 2.520(1.699~3.739) | <0.001 |
| T1-2N0M1 | 139 | 2.371(1.479~3.800) | <0.001 | 1.982(1.231~3.190) | 0.005 | 1.851(1.114~3.075) | 0.017 | 1.511(0.897~2.544) | 0.121 |
| T1-2N1M1 | 56 | 1.098(0.468~2.574) | 0.829 | 1.160(0.495~2.721) | 0.732 | 1.027(0.405~2.603) | 0.955 | 0.938(0.368~2.395) | 0.894 |

Adjusted 1 Cox regression: cox regression for age at diagnosis, year at diagnosis,sex and race matched subtype pairs.

Adjusted 2 Cox regression: cox regression for age at diagnosis, year at diagnosis, sex, race, multifocality and histological subtypes matched subtype pairs.

Adjusted 3 Cox regression: cox regression for age at diagnosis, year at diagnosis, sex, race, multifocality and histology, surgery and radiation therapy matched subtype pairs.

* represent the p value <0.05.

Table S4 Year at diagnosis of parts of diffentiated thyroid cancer patients

| Stage at diagnosis | Total number | Diagosed at 2004-2008 | Diagosed at 2009-2013 |
| --- | --- | --- | --- |
| T3N1M0 | 498 | 186 (37.3%) | 312 (62.7%) |
| T4aN0M0 | 517 | 262 (50.7%) | 255 (49.3%) |
| T4aN1M0 | 567 | 255 (45.0%) | 312 (55.0%) |
| T4bN0M0 | 260 | 144 (55.4%) | 116 (44.6%) |
| T4bN1M0 | 337 | 173 (51.3%) | 164 (48.7%) |
| T1-2N0M1 | 139 | 56 (40.3%) | 83 (59.7%) |
| T1-2N1M1 | 56 | 27(48.2%) | 29 (51.8%) |

Table S5 The follow-up time of diffentiated thyroid cancer patients

| Stage at diagnosis | Median follow-up time (months) | Number of all-cause deaths |
| --- | --- | --- |
| T3N0M0 | 43 | 254 |
| T3N1M0 | 36 | 93 |
| T4aN0M0 | 47 | 118 |
| T4aN1M0 | 37 | 170 |
| T4bN0M0 | 41 | 96 |
| T4bN1M0 | 30 | 136 |
| T1-2N0M1 | 33 | 48 |
| T1-2N1M1 | 40.5 | 15 |
| T3-4N0M1 | 19 | 86 |
| T3-4N1M1 | 15 | 184 |

Table S6: Measures of the cancer specific deaths of diffentiated thyroid cancer

|  | | Total Number | Cancer-Specific Mortality | % | Cancer-Specific Mortality | 95%CI |
| --- | --- | --- | --- | --- | --- | --- |
|  |  |  | No. |  | 1,000 Person-Years |  |
| Radiation | None or refused | 17353 | 258 | 1.5 | 3.460 | 3.036~3.943 |
|  | Radiation beam or Radioactive implants | 695 | 164 | 23.6 | 71.022 | 60.827~82.925 |
|  | Radioisotopes or Radiation beam plus isotopes or implants | 11559 | 264 | 2.3 | 5.476 | 4.853~6.180 |

Figure S1: Kaplan Meier curves between older DTC patients with T1-2N1M1 stage and patients with T3N1M0 stage (A), T4aN0M0 stage (B), T4aN1M0 stage (C), T4bN0M0 stage (D), T4bN1M0 stage (E), T1-2N0M1 stage (F) for cancer-specific survival after propensity score matched for age at diagnosis, year at diagnosis, sex and race.


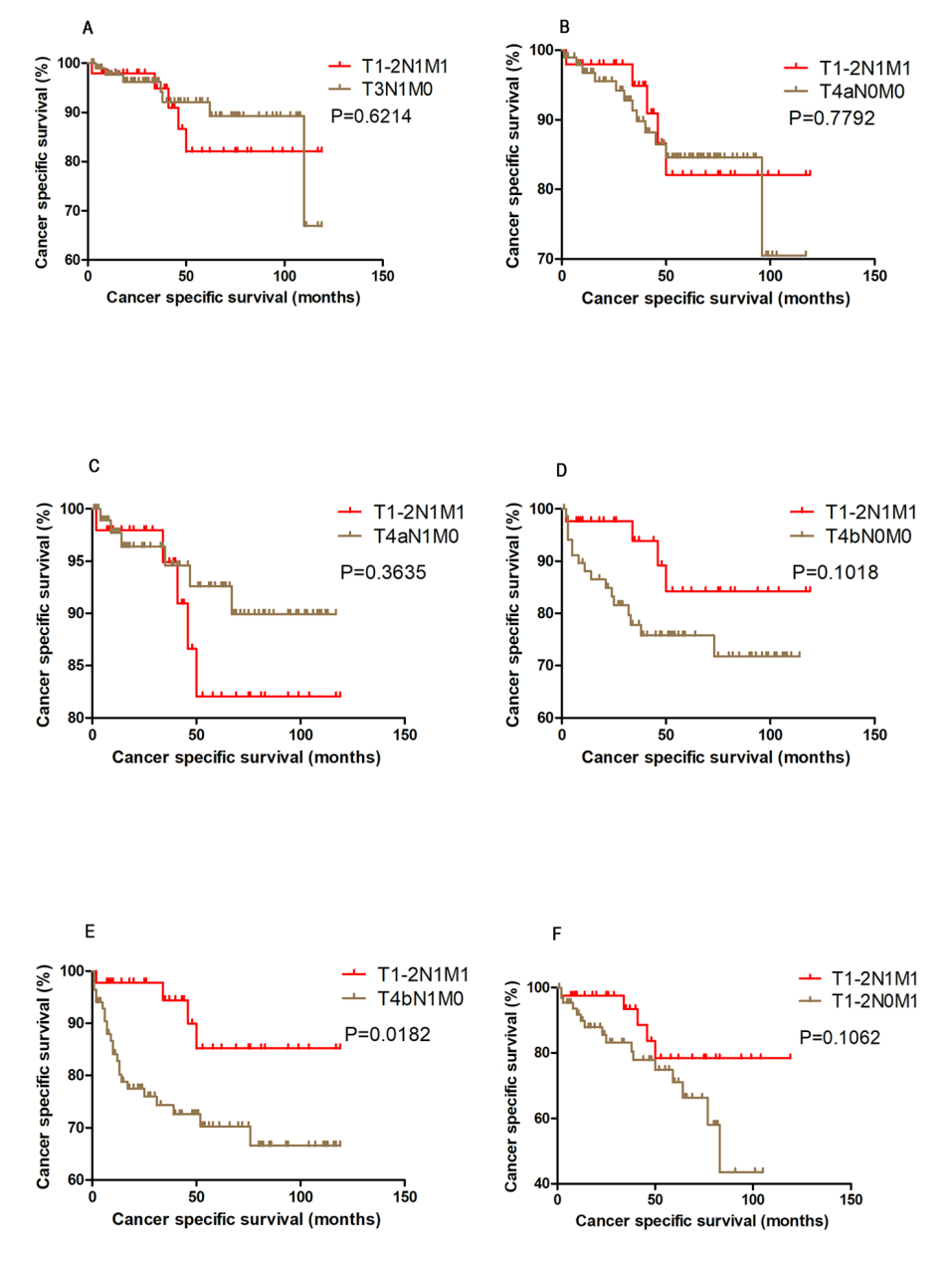


Figure S2: Kaplan Meier curves between older DTC patients with T1-2N1M1 stage and patients with T3N1M0 stage (A), T4aN0M0 stage (B), T4aN1M0 stage (C), T4bN0M0 stage (D), T4bN1M0 stage (E), T1-2N0M1 stage (F) for all-cause survival after propensity score matched for age at diagnosis, year at diagnosis, sex and race.


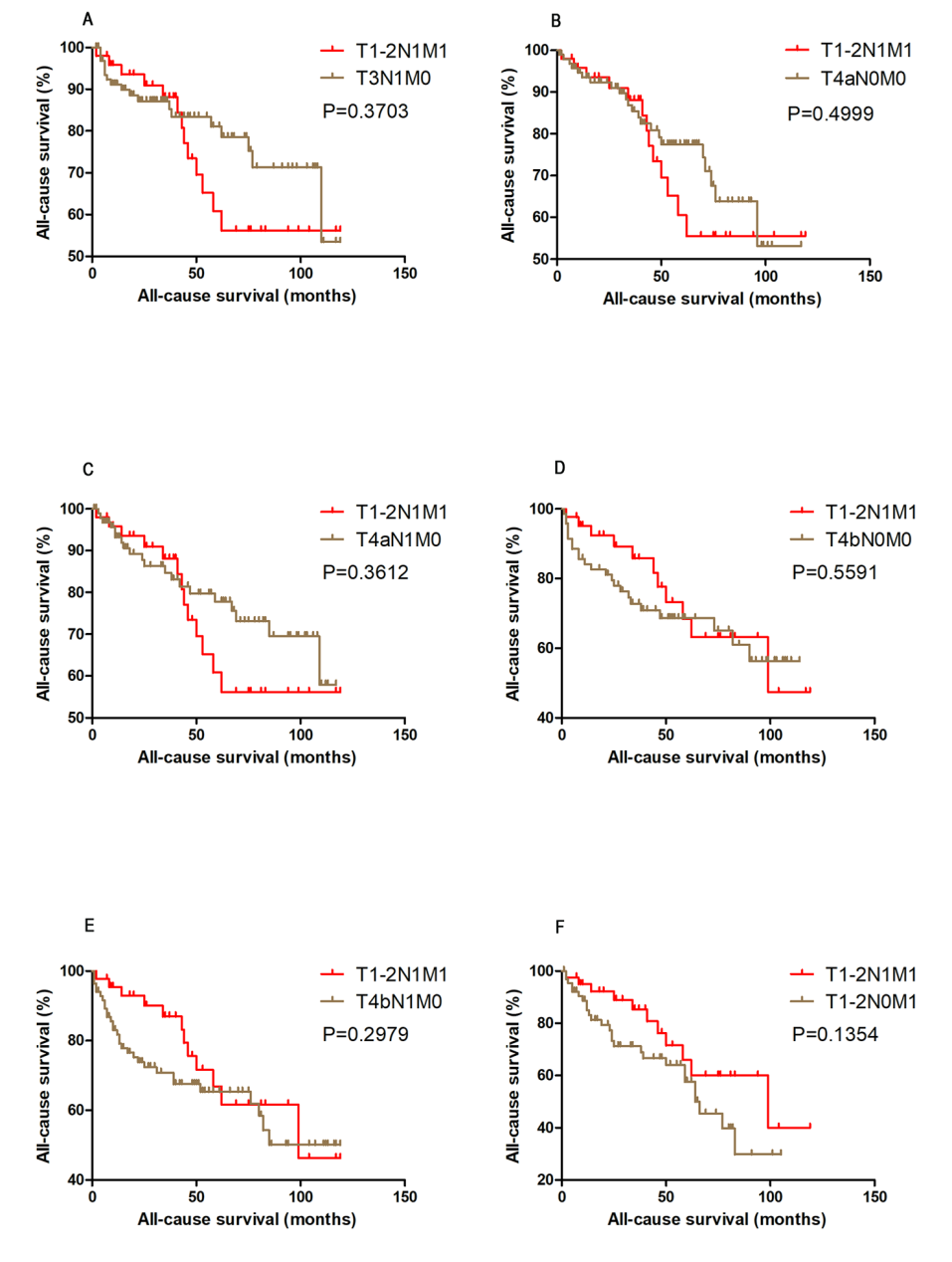


Figure S3: Kaplan Meier curves between older DTC patients with T1-2N1M1 stage and patients with T3N1M0 stage (A), T4aN0M0 stage (B), T4aN1M0 stage (C), T4bN0M0 stage (D), T4bN1M0 stage (E), T1-2N0M1 stage (F) for cancer-specific survival after propensity score matched for age at diagnosis, year at diagnosis, sex, race, multifocality and histological subtypes.


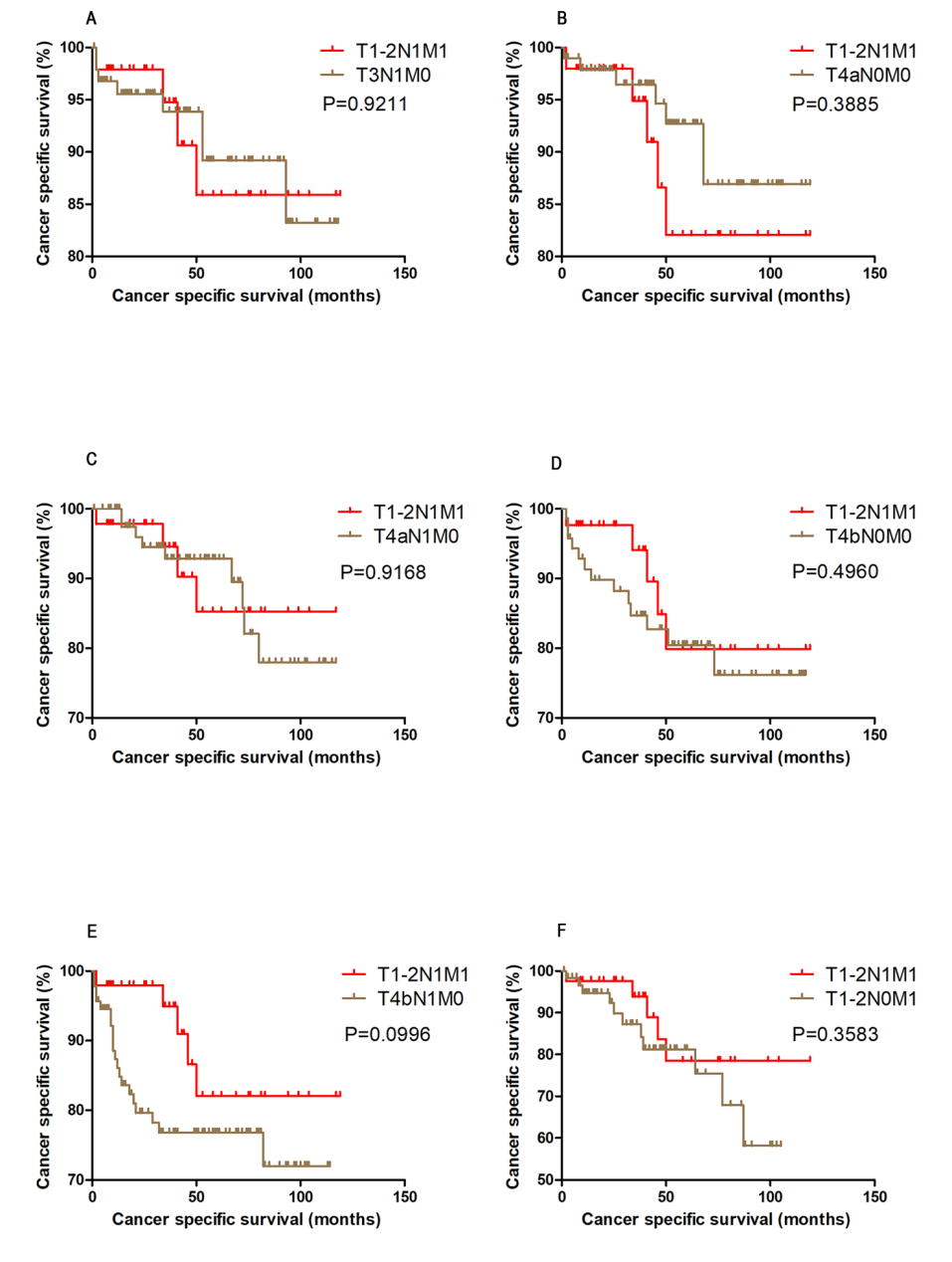


Figure S4: Kaplan Meier curves between older DTC patients with T1-2N1M1 stage and patients with T3N1M0 stage (A), T4aN0M0 stage (B), T4aN1M0 stage (C), T4bN0M0 stage (D), T4bN1M0 stage (E), T1-2N0M1 stage (F) for all-cause survival after propensity score matched for age at diagnosis, year at diagnosis, sex, race, multifocality and histological subtypes.


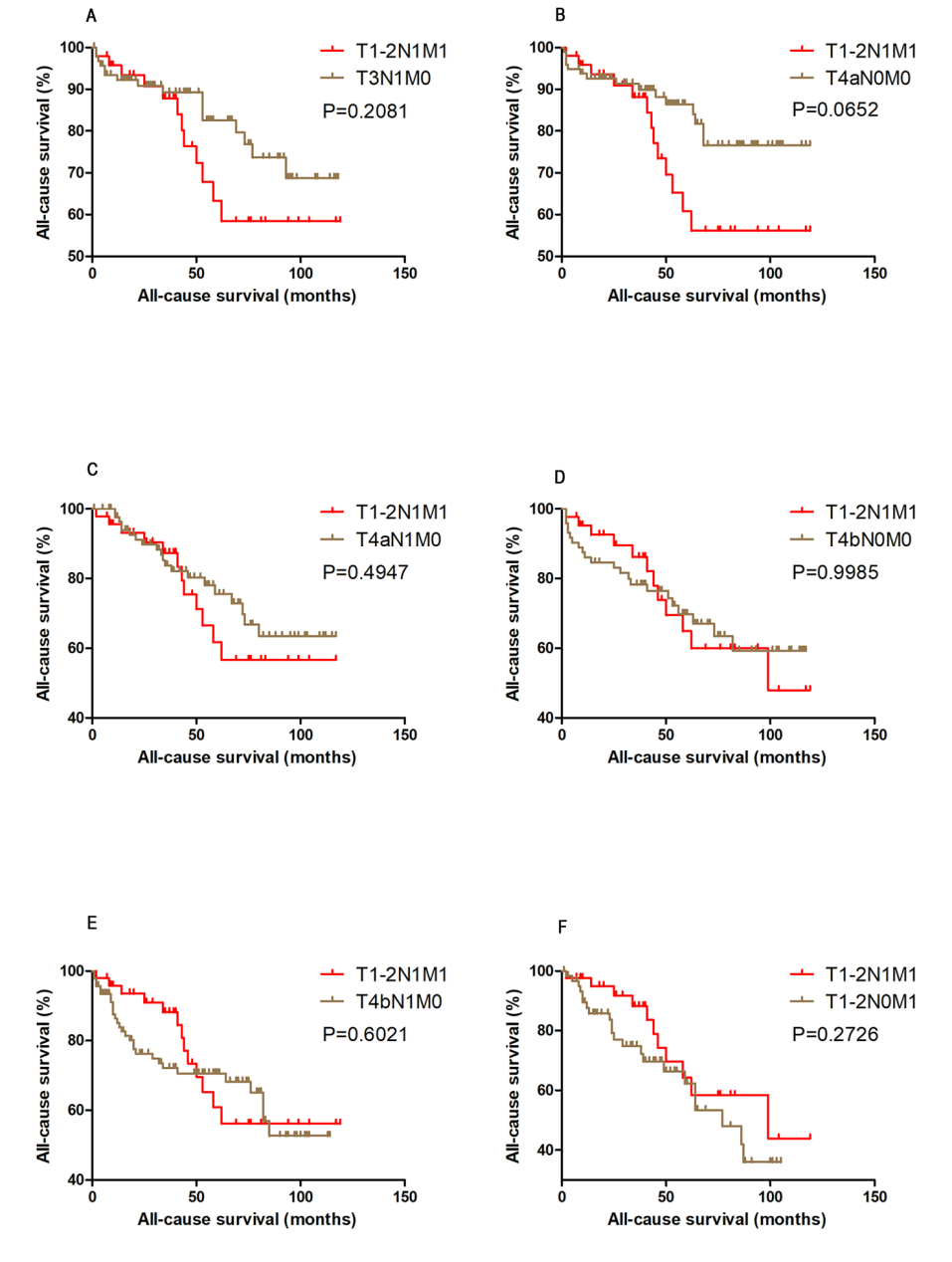


Figure S5: Kaplan Meier curves between older DTC patients with T1-2N1M1 stage and patients with T3N1M0 stage (A), T4aN0M0 stage (B), T4aN1M0 stage (C), T4bN0M0 stage (D), T4bN1M0 stage (E), T1-2N0M1 stage (F) for cancer-specific survival after propensity score matched forage at diagnosis, year at diagnosis, sex, race, multifocality, histological subtypes, surgery and radiation.


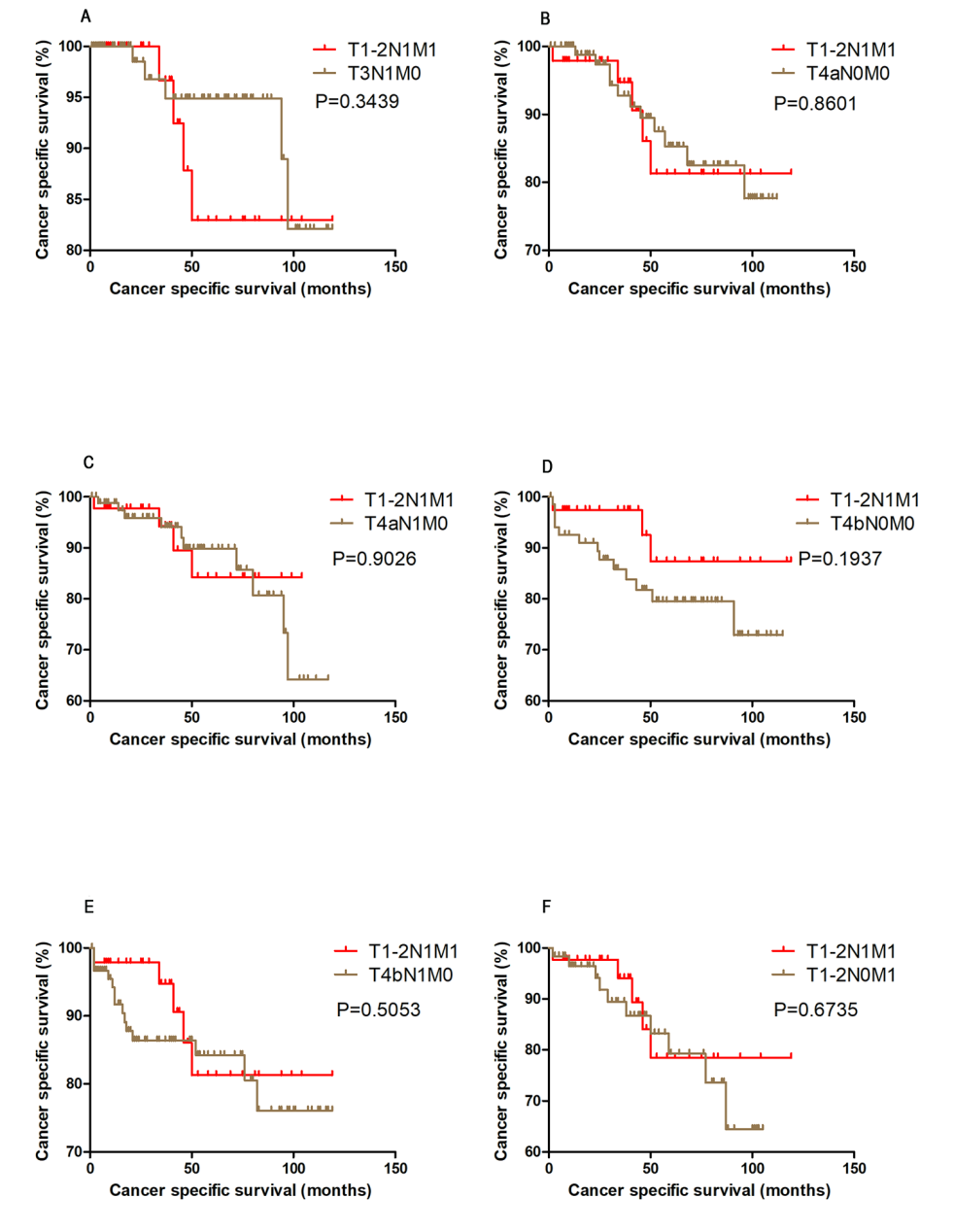


Figure S6: Kaplan Meier curves between older DTC patients with T1-2N1M1 stage and patients with T3N1M0 stage (A), T4aN0M0 stage (B), T4aN1M0 stage (C), T4bN0M0 stage (D), T4bN1M0 stage (E), T1-2N0M1 stage (F) for all-cause survival after propensity score matched for age at diagnosis, year at diagnosis, sex, race, multifocality, histological subtypes, surgery and radiation.


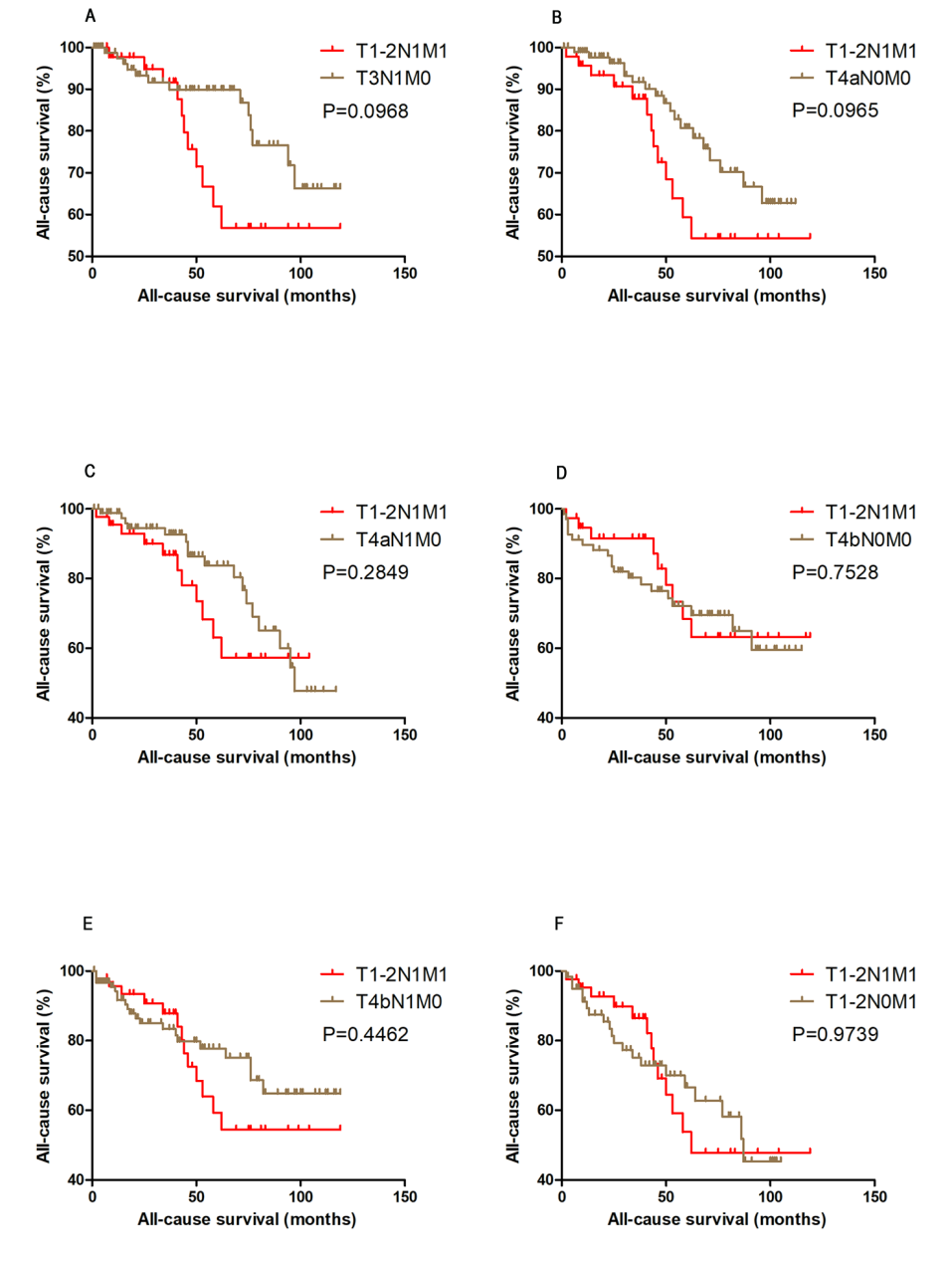


The following pictures are the relevant chapters about the cox regression analysis in the most authoritative statistical text book “Medical statistics” (the 6th edition, People’s Medical Publishing House) in China.


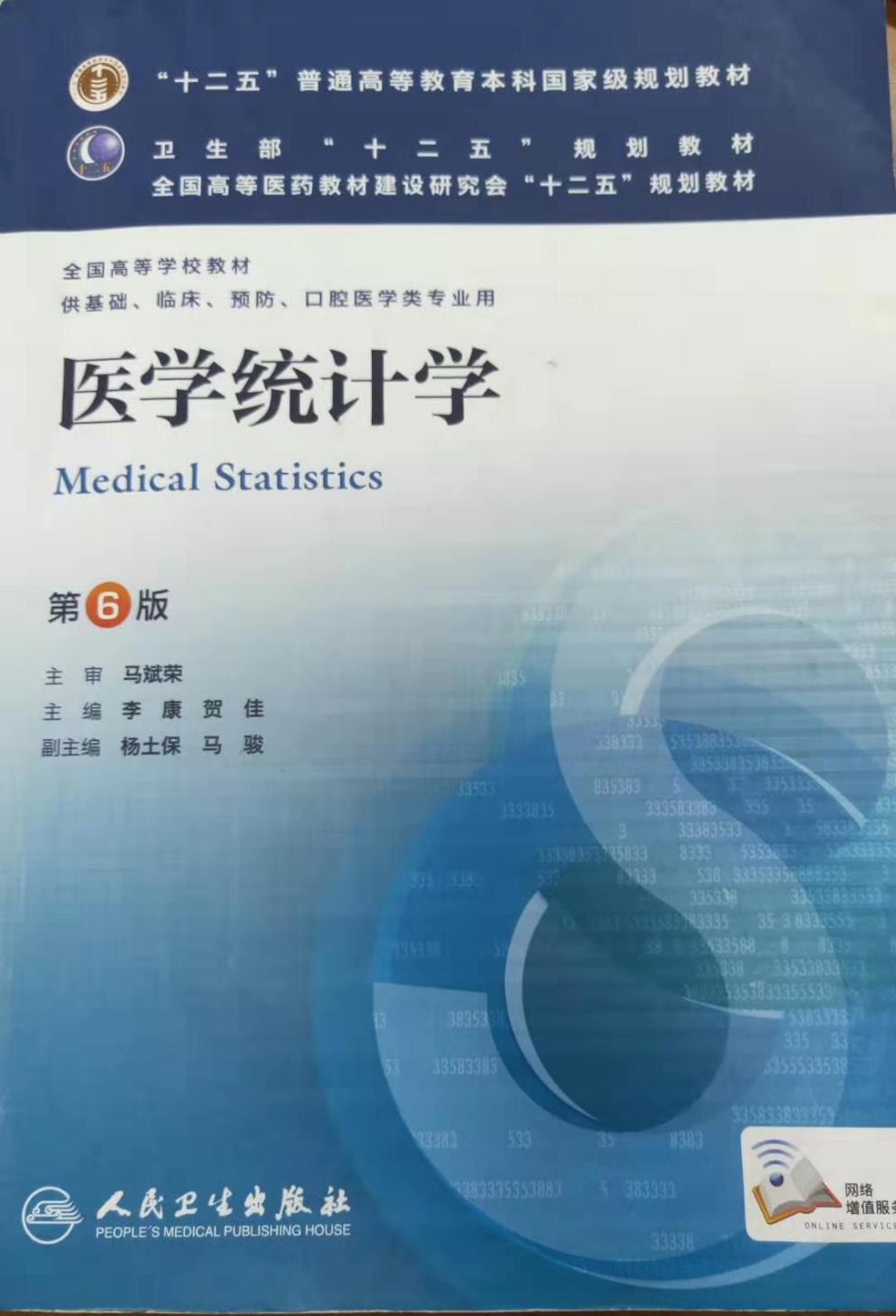


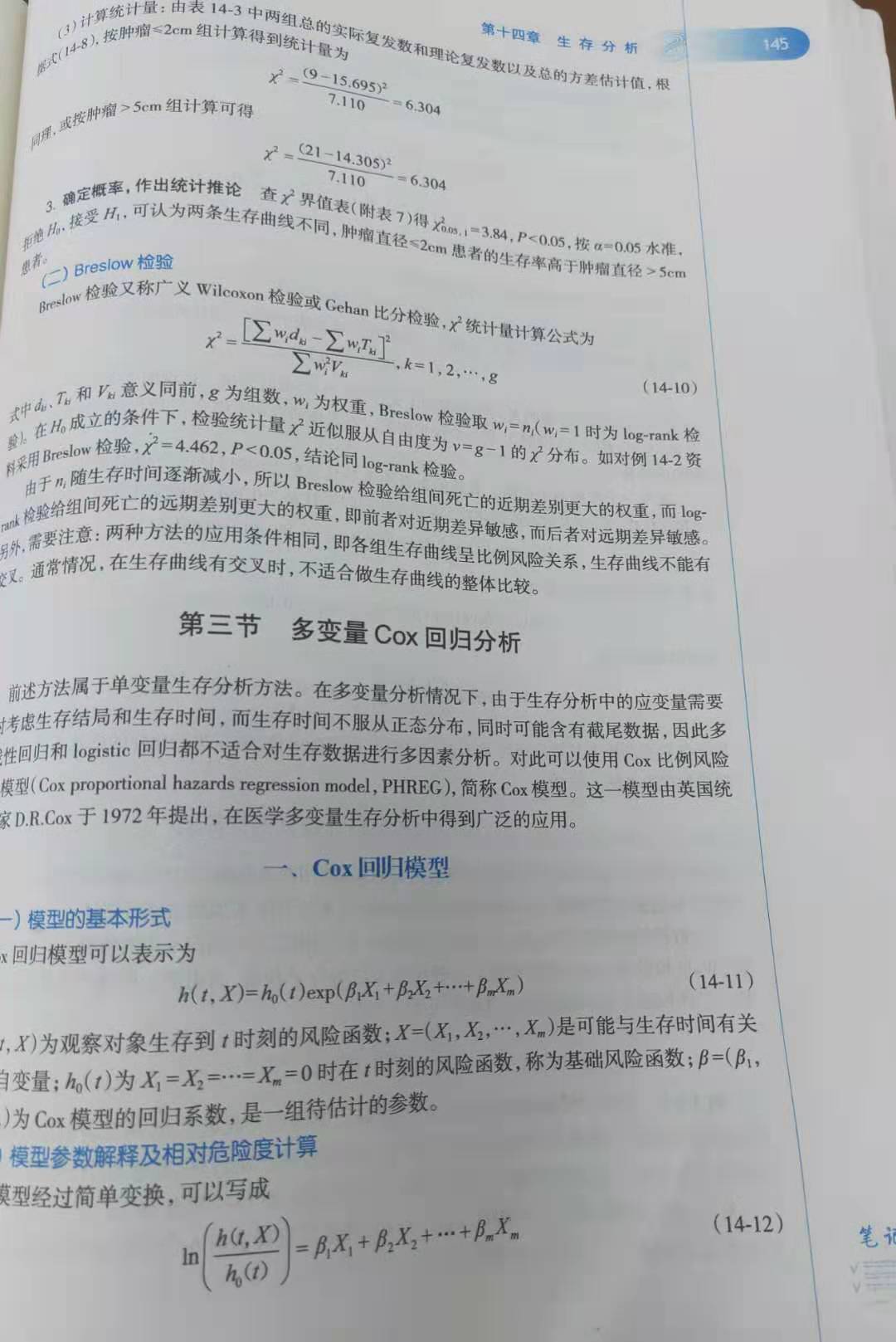

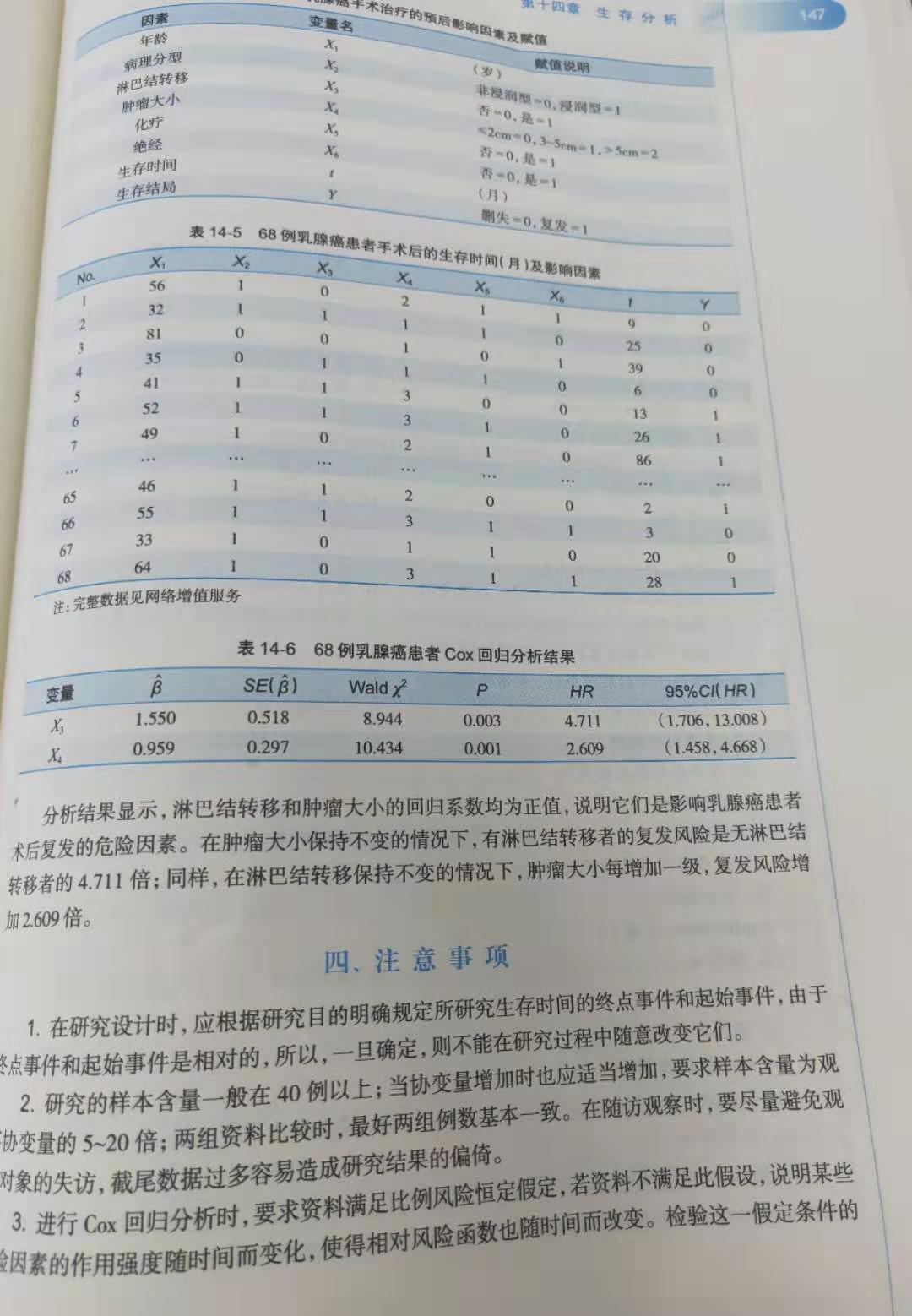


The radiation code details


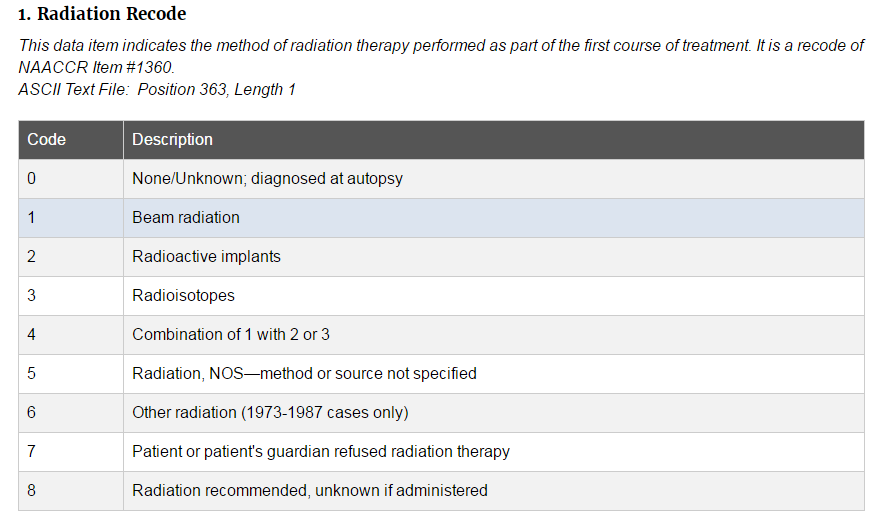


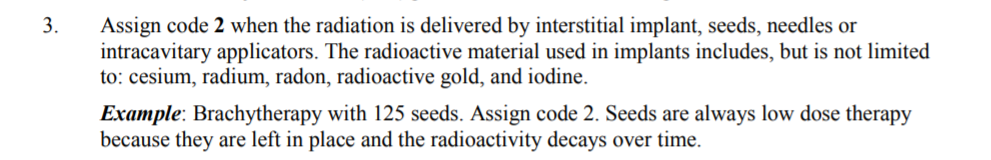

Supplement: Supplementary file 1 [file Table_1.DOCX]
